# The Use of Artificially Intelligent Self-Diagnosing Digital Platforms by the General Public: Scoping Review

**DOI:** 10.2196/13445

**Published:** 2019-05-01

**Authors:** Stephanie Aboueid, Rebecca H Liu, Binyam Negussie Desta, Ashok Chaurasia, Shanil Ebrahim

**Affiliations:** 1 Applied Health Sciences University of Waterloo Waterloo, ON Canada; 2 Faculty of Health Sciences University of Ottawa Ottawa, ON Canada; 3 Faculty of Health Sciences McMaster University Hamilton, ON Canada; 4 Meta-Research Innovation Center at Stanford Stanford University Santa Clara, CA United States

**Keywords:** diagnosis, artificial intelligence, symptom checkers, diagnostic self evaluation, self-care

## Abstract

**Background:**

Self-diagnosis is the process of diagnosing or identifying a medical condition in oneself. Artificially intelligent digital platforms for self-diagnosis are becoming widely available and are used by the general public; however, little is known about the body of knowledge surrounding this technology.

**Objective:**

The objectives of this scoping review were to (1) systematically map the extent and nature of the literature and topic areas pertaining to digital platforms that use computerized algorithms to provide users with a list of potential diagnoses and (2) identify key knowledge gaps.

**Methods:**

The following databases were searched: PubMed (Medline), Scopus, Association for Computing Machinery Digital Library, Institute of Electrical and Electronics Engineers, Google Scholar, Open Grey, and ProQuest Dissertations and Theses. The search strategy was developed and refined with the assistance of a librarian and consisted of 3 main concepts: (1) self-diagnosis; (2) digital platforms; and (3) public or patients. The search generated 2536 articles from which 217 were duplicates. Following the Tricco et al 2018 checklist, 2 researchers screened the titles and abstracts (n=2316) and full texts (n=104), independently. A total of 19 articles were included for review, and data were retrieved following a data-charting form that was pretested by the research team.

**Results:**

The included articles were mainly conducted in the United States (n=10) or the United Kingdom (n=4). Among the articles, topic areas included accuracy or correspondence with a doctor’s diagnosis (n=6), commentaries (n=2), regulation (n=3), sociological (n=2), user experience (n=2), theoretical (n=1), privacy and security (n=1), ethical (n=1), and design (n=1). Individuals who do not have access to health care and perceive to have a stigmatizing condition are more likely to use this technology. The accuracy of this technology varied substantially based on the disease examined and platform used. Women and those with higher education were more likely to choose the right diagnosis out of the potential list of diagnoses. Regulation of this technology is lacking in most parts of the world; however, they are currently under development.

**Conclusions:**

There are prominent research gaps in the literature surrounding the use of artificially intelligent self-diagnosing digital platforms. Given the variety of digital platforms and the wide array of diseases they cover, measuring accuracy is cumbersome. More research is needed to understand the user experience and inform regulations.

## Introduction

### Background

Researching health information on the internet has become common practice by the general public [1-3]. Those who do not have access to health care services are more likely to use the internet for health information [4]. In some cases, browsing the internet for health information can have certain benefits such as improving health outcomes by increasing the availability of information, providing social support, and improving self-efficacy [5,6]. However, potential negative consequences still exist; the information may not be reliable, and the individual seeking information may have low health literacy [6]. For example, an individual may not be able to critically analyze the health information and assess the applicability of the information to their case, which could result in detrimental effects on their health [6]. Therefore, health information widely circulated on the internet should be interpreted with caution [7].

Significant technological advances have resulted in the rise of more sophisticated digital health platforms, which could potentially mitigate this issue, especially those involving artificial intelligence (AI). Interest in AI appears to be relatively recent; however, the term dates back to the 1950s and is described as the theory and development of computer systems that can perform tasks that would normally require human intelligence [8,9]. Notably, AI has become incorporated in computerized diagnostic decision support systems, which were initially developed for health professionals. These platforms have now become readily available to the general public and are known as *self-diagnosing apps* or *symptom checkers*, which include the Mayo Clinic symptom checker, Babylon Health, the Ada health app, and the K Health app. On the basis of the medical information and symptoms provided by an individual, these digital platforms perform 2 main functions: (1) provide individuals with a list of potential diagnoses and (2) assist with triage [10]. While the accuracy of symptom checkers is still under question [11,12], this technology has been gaining traction globally [13,14] owing to its potential in addressing the lack of access to primary care providers (PCPs) and unnecessary medical visits—prominent issues in Canada and most parts of the world [15-18].

### Objectives

Although accuracy is important to consider, it is of equal importance to understand the overall body of knowledge that surrounds this technology, including legal and ethical implications and user experiences. In light of this, it is imperative to systematically map the literature available on artificially intelligent self-diagnosing digital platforms to identify the areas of research pertaining to this topic and to outline the key gaps in knowledge. This information can support the growing interest in leveraging AI technology in health care systems. As such, this scoping review aimed to answer the following question: What is known about the use of artificially intelligent self-diagnosing digital platforms by the general public and what are the main knowledge gaps in the literature?

## Methods

### Eligibility Criteria

In this review, self-diagnosing digital platforms were defined as platforms that utilize algorithms to provide a list of potential diagnoses to the user based on the medical information and symptoms provided. Although this scoping review does not entail quality assessment, it follows a sound methodological approach to map out the results in a concise manner for knowledge users. This scoping review follows the 2018 checklist developed by Tricco et al [19] for reporting scoping reviews. Ethics approval was not required.

The 3 main overarching concepts that guided this search were (1) self-diagnosis; (2) digital platforms; and (3) public or patients. Given the relatively new emergence of this technology and its use by the general public, the search was not limited by a publication date. Articles that were included in the review were those that (1) pertained to the use of self-diagnosing digital platforms by the lay public or patients and (2) were written in English or French. Exclusion criteria were articles that (1) focused on the use of self-diagnosing AI technology by health professionals; (2) described the back-end development of a self-diagnosing platform (eg, neural networks and architecture); (3) focused on digital health platforms that provide general health information, advice for disease management or triage; (4) focused on a tool that entails a validated questionnaire rather than an algorithm; and/or (5) examined test kits or digital platforms requiring an image upload. To allow for a wide array of results to be included, quantitative, qualitative, and mixed-methods studies or reports were eligible for inclusion.

### Information Sources and Search

This scoping review systematically searched citation databases and the gray literature for relevant published and unpublished articles. The citation databases included PubMed (Medline), Scopus, Association for Computing Machinery Digital Library, Institute of Electrical and Electronics Engineers, and Google Scholar. To supplement the gray literature retrieved through Google Scholar [20], OpenGrey and ProQuest Dissertations and Theses were also searched. The final search strategy for each data source was defined and refined with the assistance of a librarian (Rebecca Hutchinson, University of Waterloo) and was finalized on November 19, 2018. The final search strategy for PubMed (Medline) can be found in Multimedia Appendix 1. The final search results were exported into RefWorks for screening.

### Selection of Sources of Evidence

**Figure 1:**
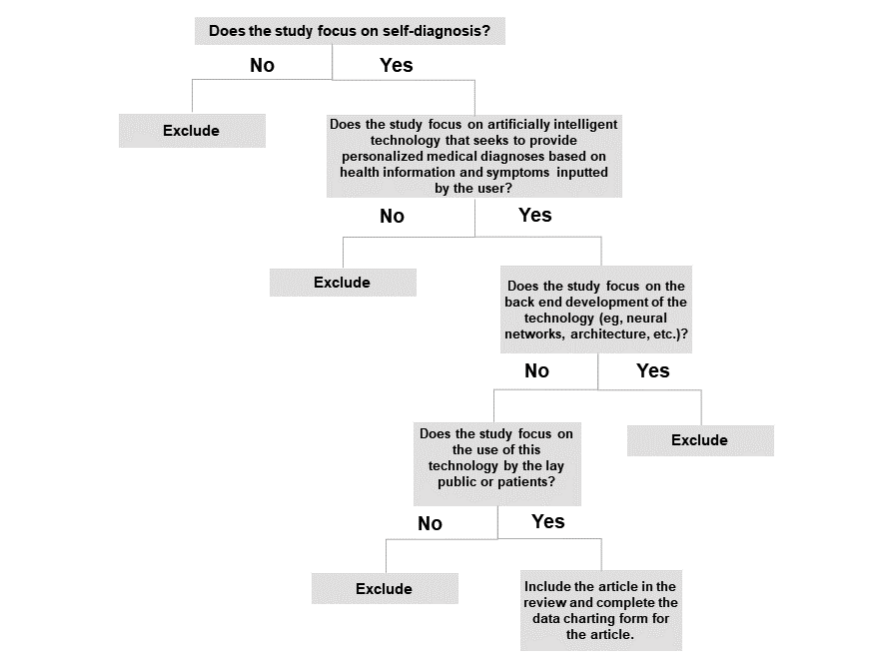
Decision tree for assessing article eligibility.

Once duplicates were removed in RefWorks, the screening process was conducted independently by 2 researchers (SA and RHL). The decision tree in Figure 1 was used as a guide to screen titles and abstracts (or executive summaries for reports and commentaries). Articles that were extracted from the title and abstract screening stage were read in their entirety (full-text review). For the full-text screening step, 2 researchers (SA and RHL) screened the same 30 articles to assess inter-rater reliability. Any uncertainty and disagreements were discussed and resolved through consensus. Following full-text review, the reference lists of eligible articles were systematically screened. Similarly, for any review paper screened at the full-text review stage, references were screened for potentially relevant articles meeting the inclusion criteria.

### Data Charting Process

Once the final number of articles was determined, a scan through these articles allowed the research team to gain a high-level understanding of the topics of interest in which self-diagnosing digital platforms were being examined (eg, accuracy and regulatory concerns). This allowed for the development of a data-charting form that captured all the relevant information, irrespective of the article type (eg, clinical trial or a qualitative study on user experience). The data-charting form was pretested with the same 5 articles to assess consistency. No changes were made to the form following this exercise.

### Data Extraction

The variables collected through the data-charting form included the following: country, year of publication, main objective, the main area of study (eg, clinical, legal, and ethical), study design, data sources used (if any), target population (if any), sample size and sample characteristics (if any), methods/statistical analyses (if applicable), main findings, and study limitations (if applicable).

### Synthesis of Results

Scoping reviews provide knowledge users with a concise overview on the literature available on a given topic of interest [21]. Given the heterogeneity of the studies included in this review, studies were grouped based on a specific area of study. A concept map was used to illustrate the breadth of studies surrounding self-diagnosing AI technology. Tables were used to provide an overview on the types of articles found in the literature and the data extracted from each article. A thematic synthesis was used to outline the knowledge gaps in the literature and other key considerations.

## Results

### Selection of Sources of Evidence

**Figure 2:**
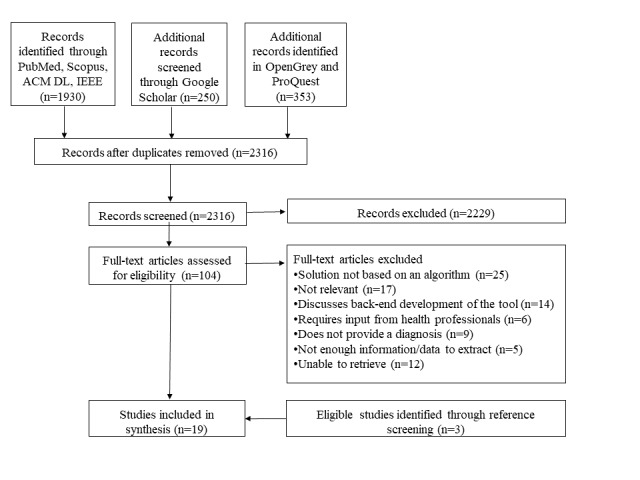
Preferred Reporting Items for Systematic Reviews and Meta-Analyses flowchart of included articles. ACM DL: Association for Computing Machinery Digital Library; IEEE: Institute of Electrical and Electronics Engineers.

Figure 2 depicts the flow chart, which illustrates the selection process at each screening step. Our search identified a total of 2536 from which 217 were duplicates. In addition, 2 researchers independently screened the titles and abstracts of 2316 articles from which 2229 were excluded based on relevance and eligibility criteria. A total of 104 full-text articles were retrieved and assessed for eligibility. Of these, 76 articles were excluded for the following reasons: described the back-end development of the digital platform or the algorithm, examined the use of digitized questionnaires rather than algorithm-based digital platforms, the digital platform required the input of health professionals, provided the risk of disease, monitored symptoms, technology designed for health professionals, not in scope, and did not provide enough data or information. We excluded 12 additional articles because we were unable to retrieve them. Through reference screening of the included articles, we identified 17 potentially relevant articles from which 3 articles were included in the review. A total of 19 articles were considered eligible for this review. Inter-rater reliability was assessed at the full-text stage which resulted in a score of 0.82, an almost perfect agreement score, between the 2 reviewers (SA and RHL) [22,23].

### Characteristics of Sources of Evidence

**Figure 3:**
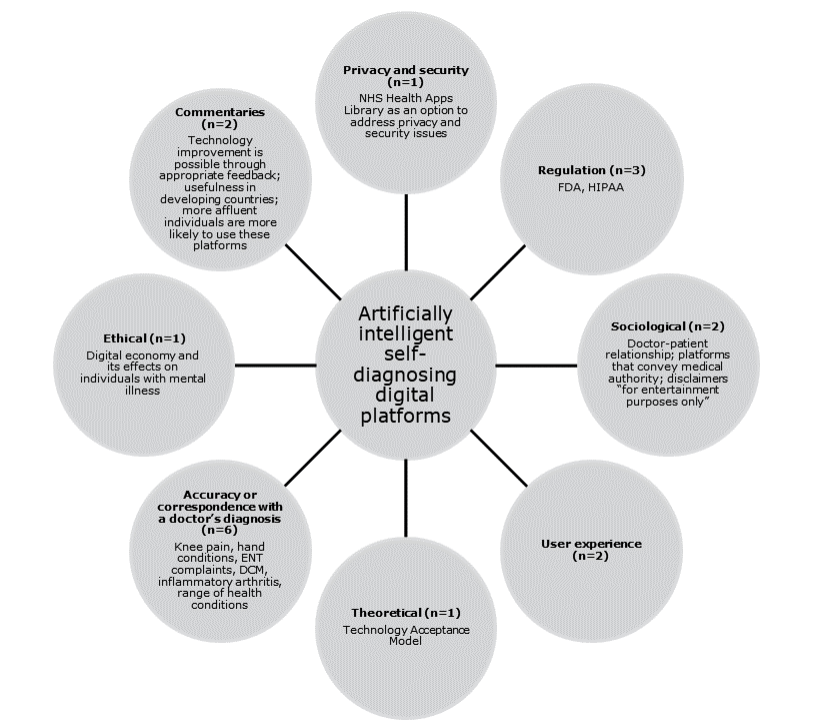
Concept map of the literature surrounding the use of artificially intelligent self-diagnosing digital platforms by the general public. DCM: degenerative cervical myelopathy; ENT: ear, nose, and throat; FDA: Food and Drug Administration; HIPAA: Health Insurance Portability and Accountability Act; NHS: National Health Service.

The concept map in Figure 3 provides an illustrative overview of the main topic areas surrounding the use of artificially intelligent self-diagnosing digital platforms by the general public. The articles were mainly conducted in the United States (n=10) or the United Kingdom (n=4). In total, 2 of the articles were commentaries and the rest focused on the following areas: accuracy or correspondence with a doctor’s diagnosis, regulation, sociological perspectives, experience, theory, privacy and security, ethics, and design. The concept map also outlines the main themes that emerged from the articles and the health conditions examined.

### Results of Individual Sources of Evidence

Multimedia Appendix 2 provides an overview of all included articles and outlines the following variables: the article type, topic area examined, main objective, and main findings [24-42].

### Synthesis of Results

Table 1 provides additional information on studies that entailed participant recruitment to answer their research question. These articles tended to focus on accuracy of the digital platform or user experience.

**Table 1:** Synthesis of results of studies with participants.

First author, year, reference, country	Sample size (n)	Target population	Data collection	Digital platforms used	Methods
Bisson, 2014 [26], United States	572	Individuals with knee pain	Primary data collection from patients and electronic medical records (EMRs)	A Web-based program developed by the research team	Sensitivity and specificity of the program’s ability to provide a correct diagnosis for knee pain was tested, out of a possible 21 conditions in which the algorithm was trained to diagnose
Bisson, 2016 [27], United States	328	Individuals with knee pain	Primary data collection from patients and EMRs	A Web-based program developed by the research team	Sensitivity and specificity were calculated
Copeland, 2018 [29], United States	13	Users who tested the protocol (specifics not provided)	Primary data collection using the System Usability Scale and the Usability Metric for User Experience	Prototype developed by the research team	Descriptive statistics
Farmer, 2011 [32], United Kingdom	61	Patients coming in to the Ear, Nose, Throat surgeon’s office	Primary data collected from patients over 1 month	Boots WebMD Symptom	Not provided
Hageman, 2014 [33], United States	86	Patients coming in to an outpatient hand and upper extremity surgeon’s office	Primary data collection from patients and physicians	WebMD Symptom Checker	The Pearson chi-square test was used to determine the level of correspondence of the provided diagnosis by the diagnostic application and the final diagnosis of the physician
Lanseng, 2007 [36], Norway	160	Individuals between the ages of 18 and 65 years	Primary data collection using the Technology Readiness Survey (TRI)	N/A^a^	A survey with an internet‐based medical self‐diagnosis application as the focal technology was conducted; The research hypotheses were tested by completing a scenario and then following-up with a questionnaire
Luger, 2014 [37], United States	79	Older adults (aged 50 years or older)	Primary data collection of think-aloud protocols	WebMD Symptom Checker	Participants received one of 2 vignettes that depicted symptoms of illness. Participants talked out loud about their thoughts and actions while attempting to diagnose the symptoms with and without the help of common internet tools (Google and WebMD’s Symptom Checker); Think-aloud content of participants was then compared with those who were accurate in their diagnosis versus those who were not.
Powley, 2016 [40], United Kingdom	34	Consecutive patients with newly presenting clinically apparent synovitis or a new onset of symptoms consistent with inflammatory arthritis	Primary data collection from patients	National Health Service (NHS) and WebMD Symptom Checkers	Patients were asked questions about their internet use in relation to their presenting symptoms. Subsequently, they completed the NHS and the WebMD symptom checkers and their answers as well as outcomes were recorded.

^a^Not applicable.

## Discussion

### Summary of Evidence and Knowledge Gaps

In this scoping review, 19 articles were included that examined artificially intelligent self-diagnosing digital platforms from various perspectives. Despite the popularity and accessibility of self-diagnosing AI technology by the public, it is noteworthy that research examining the accuracy of these platforms is limited. As such, it is unclear whether these platforms hinder or improve the health of users. Although some argue that the use of this technology may cause an individual to delay seeking care, it is important to recognize that delayed diagnoses are prevalent even without the use of this technology [40,42,43]. Many factors contribute to a delayed diagnosis with the top-ranked issues being poor communication between secondary and primary care, a mismatch between patients’ medical needs and health care supply, and a lack of access or use of health services [42,44]. For example, Behrbalk et al found that the average time delay from initiation of symptoms to the diagnosis of cervical spondylotic myelopathy (CSM) was 2.2 (SD 2.3) years [43]. Although symptom checkers can potentially address delayed diagnoses, a review showed that this technology was suboptimal in diagnosing CSM [30].

Moreover, these platforms generally provide a list of potential diagnoses rather than a single diagnosis. In this case, the user must decide which condition describes their current state best. The likelihood of a user to accurately choose the right diagnosis is associated with the sociodemographic profile/variables of a user, such as education and gender [33]. For example, women and those with higher education were more likely to choose the correct diagnosis [33]. Therefore, although having a timely diagnosis is important, it may be counterproductive if the user considers the wrong treatment options owing to a misdiagnosis. Moreover, the patient may still require a visit to a PCP to receive treatment or a prescription. Issues may arise if patients already have a diagnosis in mind when visiting their PCP as it could translate into disagreements regarding their condition.

This scoping review suggests that there are prominent knowledge gaps in the literature; as such, a systematic review may not be worthwhile on this topic. Rather, concerted efforts are needed in producing research in this area related to accuracy, user experience, regulation, doctor-patient relationship, PCP perspectives, and ethics. Specifically, extensive research is needed in evaluating the accuracy of this technology while accounting for the fact that some platforms are designed for a wide area of conditions and others are specialized—as such, these platforms need to be evaluated accordingly. It is also important to distinguish the difference between accuracy and correspondence with a PCP’s diagnosis as PCPs may misdiagnose or miss a diagnosis [45-47]. Importantly, when developing self-diagnosing AI digital platforms, it is important to test them on users with a wide range of backgrounds and level of experience with technology. This will ensure that a high proportion of users will end up choosing the right diagnosis.

Along with the importance of accuracy in self-diagnosing applications, there also needs to be guidance on how these platforms should be regulated. Although regulations related to self-diagnosing AI technologies should focus on patient safety as well as privacy and security, they should not hinder innovation in this area; rather, they should allow innovative advancements that are safe and improve access to timely diagnosis. Overall, more knowledge is needed on how different types of users interact with this technology and how its use can impact the PCP-patient relationship. There is also a need for clarity on data management shared by users. Ethical concerns surrounding the digital economy is a main area of concern, and there is currently a debate surrounding the trade-offs pertaining to the use of these platforms.

### Limitations

Some limitations of this scoping review warrant mention. Artificially intelligent self-diagnosing platforms that require individuals to upload an image or a scan were excluded from the review. Test kits or platforms that would require the user to perform medical tests were also excluded. Our scoping review’s focus was on platforms that required the least amount of effort from the user (ie, simply entering their symptoms into the platform to obtain potential diagnoses). It is also possible that some potentially relevant articles were missed because they could not be retrieved. To counteract this limitation, the authors systematically reviewed the references of relevant articles and held multiple meetings to assess consistency and to discuss any discrepancies in the screening process.

### Conclusions

Given self-diagnosing AI technology’s potential, it is worth understanding how it can be leveraged by health care systems to reduce costs and unnecessary medical visits. This scoping review aimed to map the literature surrounding the use of artificially intelligent self-diagnosing platforms. Given the direct-to-consumer approach of these platforms, it is worrisome that only a few studies have focused on the use of this technology. It is important that future research and resources are directed to understanding the accuracy and regulation of self-diagnosing AI digital platforms. These regulations may take different forms such as creating an application library which includes a list of platforms that have been deemed safe and provide highly accurate diagnoses from a credible health agency or organization. It should be noted that patient engagement is necessary in the development of these platforms to ensure that they allow a high proportion of individuals—irrespective of gender and education—to choose the right diagnosis. Importantly, user experience is crucial to consider as the public may be skeptical of this technology.

## Appendix

Multimedia Appendix 1 Search strategy for PubMed (Medline).

Multimedia Appendix 2 Overview of included studies related to self-diagnosing artificial intelligence digital platforms.

## Abbreviations

AI: artificial intelligence
CSM: cervical spondylotic myelopathy
PCP: primary care provider

## References

[1] Statistics Canada.

[2] Beck F, Richard JB, Nguyen-Thanh V, Montagni I, Parizot I, Renahy E (2014). Use of the internet as a health information resource among French young adults: results from a nationally representative survey. J Med Internet Res.

[3] Pew Research Centre.

[4] Amante DJ, Hogan TP, Pagoto SL, English TM, Lapane KL (2015). Access to care and use of the internet to search for health information: results from the US National Health Interview Survey. J Med Internet Res.

[5] Ybarra ML, Suman M (2006). Help seeking behavior and the internet: a national survey. Int J Med Inform.

[6] Tonsaker T, Bartlett G, Trpkov C (2014). Health information on the internet: gold mine or minefield?. Can Fam Physician.

[7] Mosa AS, Yoo I, Sheets L (2012). A systematic review of healthcare applications for smartphones. BMC Med Inform Decis Mak.

[8] Turing A (1950). Computing machinery and intelligence. Mind.

[9] Senate Canada (2017). Standing Senate Committee on Social Affairs, Science and Technology.

[10] Semigran HL, Linder JA, Gidengil C, Mehrotra A (2015). Evaluation of symptom checkers for self diagnosis and triage: audit study. Br Med J.

[11] Fraser H, Coiera E, Wong D (2018). Safety of patient-facing digital symptom checkers. Lancet.

[12] Millenson ML, Baldwin JL, Zipperer L, Singh H (2018). Beyond Dr Google: the evidence on consumer-facing digital tools for diagnosis. Diagnosis (Berl).

[13] (2018). Business Insider.

[14] (2018). Digital Health.

[15] Brownlee S, Chalkidou K, Doust J, Elshaug AG, Glasziou P, Heath I, Nagpal S, Saini V, Srivastava D, Chalmers K, Korenstein D (2017). Evidence for overuse of medical services around the world. Lancet.

[16] Morgan DJ, Dhruva SS, Wright SM, Korenstein D (2016). 2016 update on medical overuse: a systematic review. JAMA Intern Med.

[17] (2017). Canadian Institute of Health Information.

[18] (2019). Organisation for Economic Co-operation and Development.

[19] Tricco AC, Lillie E, Zarin W, O'Brien KK, Colquhoun H, Levac D, Moher D, Peters MD, Horsley T, Weeks L, Hempel S, Akl EA, Chang C, McGowan J, Stewart L, Hartling L, Aldcroft A, Wilson MG, Garritty C, Lewin S, Godfrey CM, Macdonald MT, Langlois EV, Soares-Weiser K, Moriarty J, Clifford T, Tunçalp Ö, Straus SE (2018). PRISMA Extension for Scoping Reviews (PRISMA-ScR): Checklist and Explanation. Ann Intern Med.

[20] Haddaway NR, Collins AM, Coughlin D, Kirk S (2015). The role of Google Scholar in evidence reviews and its applicability to grey literature searching. PLoS One.

[21] (2016). Canadian Institutes of Health Research.

[22] McHugh ML (2012). Interrater reliability: the kappa statistic. Biochem Med (Zagreb).

[23] Wongpakaran N, Wongpakaran T, Wedding D, Gwet KL (2013). A comparison of Cohen's kappa and Gwet's AC1 when calculating inter-rater reliability coefficients: a study conducted with personality disorder samples. BMC Med Res Methodol.

[24] Bauer M, Glenn T, Monteith S, Bauer R, Whybrow PC, Geddes J (2017). Ethical perspectives on recommending digital technology for patients with mental illness. Int J Bipolar Disord.

[25] Weldegebrial T (2016). Marshall University, Department of Health Informatics.

[26] Bisson LJ, Komm JT, Bernas GA, Fineberg MS, Marzo JM, Rauh MA, Smolinski RJ, Wind WM (2014). Accuracy of a computer-based diagnostic program for ambulatory patients with knee pain. Am J Sports Med.

[27] Bisson LJ, Komm JT, Bernas GA, Fineberg MS, Marzo JM, Rauh MA, Smolinski RJ, Wind WM (2016). How accurate are patients at diagnosing the cause of their knee pain with the help of a web-based symptom checker?. Orthop J Sports Med.

[28] Boulos MNK, Brewer AC, Karimkhani C, Buller DB, Dellavalle RP (2014). Mobile medical and health apps: state of the art, concerns, regulatory control and certification. Online J Public Health Inform.

[29] Copeland C, Morreale P, Li J (2018). m-Health application Interface design for symptom checking. http://tinyurl.com/y5jotoby.

[30] Davies BM, Munro CF, Kotter MR (2019). A novel insight into the challenges of diagnosing degenerative cervical myelopathy using web-based symptom checkers. J Med Internet Res.

[31] Flaherty JL (2014). Digital diagnosis: privacy and the regulation of mobile phone health applications. Am J Law Med.

[32] Farmer SE, Bernardotto M, Singh V (2011). How good is internet self-diagnosis of ENT symptoms using Boots WebMD symptom checker?. Clin Otolaryngol.

[33] Hageman MG, Anderson J, Blok R, Bossen JK, Ring D (2015). Internet self-diagnosis in hand surgery. Hand (N Y).

[34] Jutel A, Lupton D (2015). Digitizing diagnosis: a review of mobile applications in the diagnostic process. Diagnosis (Berl).

[35] Kao C, Liebovitz DM (2017). Consumer mobile health apps: current state, barriers, and future directions. PM R.

[36] Lanseng E, Andreassen T (2007). Electronic healthcare: a study of people's readiness and attitude toward performing self‐diagnosis. Int J of Service Industry Mgmt.

[37] Luger TM, Houston TK, Suls J (2014). Older adult experience of online diagnosis: results from a scenario-based think-aloud protocol. J Med Internet Res.

[38] Lupton D, Jutel A (2015). 'It's like having a physician in your pocket!' A critical analysis of self-diagnosis smartphone apps. Soc Sci Med.

[39] Morita T, Rahman A, Hasegawa T, Ozaki A, Tanimoto T (2017). The potential possibility of symptom checker. Int J Health Policy Manag.

[40] Powley L, McIlroy G, Simons G, Raza K (2016). Are online symptoms checkers useful for patients with inflammatory arthritis?. BMC Musculoskelet Disord.

[41] Ryan A, Wilson S (2008). Internet healthcare: do self-diagnosis sites do more harm than good?. Expert Opin Drug Saf.

[42] Tudor Car L, Papachristou N, Bull A, Majeed A, Gallagher J, El-Khatib M, Aylin P, Rudan I, Atun R, Car J, Vincent C (2016). Clinician-identified problems and solutions for delayed diagnosis in primary care: a PRIORITIZE study. BMC Fam Pract.

[43] Behrbalk E, Salame K, Regev GJ, Keynan O, Boszczyk B, Lidar Z (2013). Delayed diagnosis of cervical spondylotic myelopathy by primary care physicians. Neurosurg Focus.

[44] Aboueid S, Meyer SB (2019). Factors affecting access and use of preventive and weight management care: A public health lens. Healthc Manage Forum.

[45] Singh H, Schiff GD, Graber ML, Onakpoya I, Thompson MJ (2017). The global burden of diagnostic errors in primary care. BMJ Qual Saf.

[46] Panesar SS, deSilva D, Carson-Stevens A, Cresswell KM, Salvilla SA, Slight SP, Javad S, Netuveli G, Larizgoitia I, Donaldson LJ, Bates DW, Sheikh A (2016). How safe is primary care? A systematic review. BMJ Qual Saf.

[47] Nurek M, Kostopoulou O, Delaney BC, Esmail A (2015). Reducing diagnostic errors in primary care. A systematic meta-review of computerized diagnostic decision support systems by the LINNEAUS collaboration on patient safety in primary care. Eur J Gen Pract.

