# Supplementary material for: The Use of Artificially Intelligent Self-Diagnosing Digital Platforms by the General Public: Scoping Review
Source: JMIR Med Inform. 2019 May 1;7(2):e13445. doi: 10.2196/13445 (PMC6658267; doi:10.2196/13445)
Supplement: Multimedia Appendix 1 [file medinform_v7i2e13445_app1.pdf]

Pubmed Search Strategy

Step 1. Isolate the main concepts of the research topic. (Add/subtract columns as needed). List all relevant search terms for each concept. Include author keywords and subject headings (i.e. MeSH headings)

**Concept 1**	**Concept 2**	**Concept 3**
Self diagnosing[tiab] OR self diagnosis[tiab] OR self evaluation*[tiab] OR self appraisal*[tiab] OR symptom check*[tiab] OR check your symptom*[tiab] OR check their symptom*[tiab] OR self triage[tiab] OR self-triage [tiab]	Technolog*[tiab] OR technology[mesh:noexp] OR website*[tiab] OR online[tiab] OR computing methodologies[mesh] OR computer* [tiab] OR algorithm*[tiab] OR mhealth[tiab] OR m-health [tiab] OR ehealth[tiab] OR e-health [tiab] OR app[tiab] OR apps[tiab] OR mobile application[tiab] OR smartphone[mesh] OR smart phone*[tiab] OR smartphone*[tiab] OR cell phone*[tiab] OR cellular phone*[tiab] OR mobile phone*	Population [tiab] OR person*[tiab] OR patient [tiab] OR patients [tiab] OR individual*[tiab] OR consumer* [tiab] OR people [tiab] OR patients[mesh:noexp]

Step 2. Translate the above list into a search statement. Create one search strategy rather than multiple searches. Combine the search terms using AND/OR and brackets ( ). Use Line #s to organize longer searches.

((((Self diagnosing[tiab] OR self diagnosis[tiab] OR self evaluation*[tiab] OR self appraisal*[tiab] OR symptom check*[tiab] OR check your symptom*[tiab] OR check their symptom*[tiab] OR self triage[tiab] OR self-triage [tiab]) AND ((English[lang] OR French[lang])))) AND ((Technolog*[tiab] OR technology[mesh:noexp] OR website*[tiab] OR online[tiab] OR computing methodologies[mesh] OR computer* [tiab] OR algorithm*[tiab] OR mhealth[tiab] OR m-health [tiab] OR ehealth[tiab] OR e-health [tiab] OR app[tiab] OR apps[tiab] OR mobile application[tiab] OR smartphone[mesh] OR smart phone*[tiab] OR smartphone*[tiab] OR cell phone*[tiab] OR cellular phone*[tiab] OR mobile phone*) AND ((English[lang] OR French[lang])))) AND ((Population [tiab] OR person*[tiab] OR patient [tiab] OR patients [tiab] OR individual*[tiab] OR consumer* [tiab] OR people [tiab] OR patients[mesh:noexp]) AND ((English[lang] OR French[lang])))

Step 3. Retrieve articles (n=611)

Step 4. Filter by language: English and French
