# Supplementary material for: The Use of Artificially Intelligent Self-Diagnosing Digital Platforms by the General Public: Scoping Review
Source: JMIR Med Inform. 2019 May 1;7(2):e13445. doi: 10.2196/13445 (PMC6658267; doi:10.2196/13445)
Supplement: Multimedia Appendix 2 [file medinform_v7i2e13445_app2.pdf]

Table 1. Overview of included studies related to self-diagnosing AI digital platforms.

First Author (Year) [Reference]	Article type, Topic area	Main objective	Main findings/discussion points
Bauer M (2017) [24]	Review, Ethical	To increase understanding and promote discussion on the ethical issues of the digital economy that affect the treatment of patients with mental illness	Privacy is rarely guaranteed; There are societal pressures to disclose personal information; Usage of invalidated apps involve medical risks; Physicians should be aware of these apps to guide patients appropriately
Weldegebrial T (2016) [25]	Review, Legal	To examine the FDA[1] and HIPAA[2] regulations for health care apps and to suggest additional regulation requirements that could be used globally	Many health apps are unregulated by the FDA and HIPPA; Enforcing strict regulations might stifle innovation of beneficial apps
Bisson L (2014) [26]	Comparative, Accuracy	To design and evaluate an internet-based program that generates a differential diagnosis based on a history of knee pain entered by the patient	High sensitivity corresponding to correct knee pain diagnosis; Low specificity is expected owing to differential diagnosis; Missed diagnosis owing to program limitation, error, and incorrect inputs by users
Bisson L (2018) [27]	Comparative, Accuracy	To evaluate a patient's ability to self-diagnose their knee pain from a list of possible diagnoses	Patients were able to correctly identify the cause of their knee pain in 315 out of 543 cases (ie, 58% of the time); The accuracy of a program to

		supplied by a symptom checker	generate a diagnosis may not be able to be improved without the ability to gather data from a physical examination
Boulos M (2014) [28]	Narrative, Legal/Regulation	To describe the range of apps on offer as of 2013 and then present a brief survey of evaluation studies of medical and health-related apps that were conducted to date, covering a range of disciplines and topics	App development should include medical experts and requires maintenance and regular updating which may entail significant costs; Lack of regulation undermines the population's safety; To ensure user safety, education regarding the use of these apps should be a first-line solution
Copeland C (2018) [29]	Cross-sectional, User experience	To create a simple interface for a symptom checker and evaluate the design by surveying users	Mobile health (mHealth) symptom checker was well received; It works well on a modest range of common ailments; It can be used conditionally to disseminate appropriate medical information
Davies BM (2018) [30]	Cross-sectional/ Accuracy	To investigate whether online symptom checkers are able to recognize relevant symptoms of Degenerative Cervical Myelopathy (DCM) differential returned and to evaluate the	DCM symptoms perform inadequately in symptom checkers; With the required optimization, symptom checkers are still attractive; Language barrier, computer proficiency, and the algorithms are some of the hindering

		diagnostic performance of its recognized symptoms	factors limiting usability of symptom checkers
Flaherty JL (2014) [31]	Narrative/ Legal	To examine regulations in which mHealth apps such as self-diagnosing apps are subjected to and the privacy/security concerns related to them	Many apps are unregulated by FDA or HIPAA; Unclear usage of consumers' information; The regulation leniency to allow for innovation comes with information risk
Farmer SEJ (2011) [32]	Prospective/ Accuracy	To report the findings of a study that examined the accuracy of Boots WebMD symptom checker in diagnosing ENT complaints	The median number of differential diagnoses provided per patient was 13 (range 1-20); The symptom checker correctly diagnosed 43 out of 61 patients
Hageman MGJS (2014) [33]	Prospective observational study/ Correspondence of diagnosis from the symptom checker with one of the surgeons	To test the null hypothesis that there are no factors associated with correspondence between online diagnosis and the hand surgeon's diagnosis in an outpatient hand and upper extremity surgeon's office	Factors associated with a web-based diagnosis corresponding to the hand surgeon's diagnosis included sex (women) and patients who studied their symptoms online prior to the visit; Considering the uniqueness of various symptom clusters and the probability of specific disease may improve diagnosis accuracy of symptom checkers
Jutel A (2015) [34]	Review of apps/ Sociological perspective	To describe and catalogue available diagnosis apps and	The 4 app categories are diagnosis, diagnosis coding, e-documents, and medical education; These apps are

		explore their impact on the diagnostic process	improving access to medical information, but credibility is a concern
Kao CK (2017) [35]	Narrative/ Unsure	To describe the current state, barriers, and future directions of mHealth apps and eventually take the leading role to drive the change	Lack of regular supervision, limited evidence-based literature, and privacy and security concerns are the barriers to efficacy of mHealth apps; Despite the barriers, there exists potential for evolution of these apps
Lanseng EJ (2007) [36]	Cross-sectional survey/ Theoretical	To examine the introduction of self-service technology (SST) in health diagnosis as a means to reduce costs and improve quality in the health care sector at the same time	People might accept the use of self-diagnosis technology; Consumers' expectation, convenience, ease of use, and trust are the key drivers for adoption and usage of SST
Luger TM (2014) [37]	Cross-sectional (qualitative)/ User experience	To describe the processes that a sample of older adults may use to diagnose symptoms online as well as the processes that predict accurate diagnosis	Participants relied on their experience and rejected the diagnosis if it was discordant; Confusion with the process, untrusting the diagnosis, tendency to rely on past experience are reasons for inaccurate diagnosis
Lupton D (2015) [38]	Review/ Sociological perspective	To examine the ways in which self-diagnosis apps were portrayed on	Even if many apps lack a description statement, they denote a sense of authority, scientific objectivity, and

		the Apple App store and Google Play websites	accuracy; Many apps added the tag *for entertainment purpose* which may undermine their credibility
Morita T (2017) [39]	N/A[3]/ Letter to the editor	To introduce the possible benefit of symptom checkers on public health	Symptom checkers can help community health workers in resource-limited countries; With incorporation of feedback from health professionals, symptom checkers can be improved
Powley L (2016) [40]	Comparative/ Accuracy	To evaluate how patients with inflammatory arthritis and inflammatory arthralgia use the internet to look for health information and to assess the advice given and diagnoses suggested by the NHS and WebMD symptom checkers in relation to the patients' actual diagnoses	Only 4 out of 21 patients with inflammatory arthritis were given a first diagnosis of rheumatoid arthritis or psoriatic arthritis; Help-seeking advice given online is often inappropriate and the diagnoses suggested are frequently inaccurate
Ryan A (2008) [41]	Review/ Expert Opinion	To describe the possible impact of the use of self-diagnosis websites.	Affluence and higher education attainment increased the interest in self-care
Semigran HL (2015) [42]	Audit study/ Accuracy	To determine the diagnostic and triage accuracy of online symptom checkers.	Symptom checkers provided the correct diagnosis first for 262 out of 770 patient vignettes; The correct

			diagnosis was listed within the top 3 diagnoses, 394 out of 770 patient vignettes; The correct diagnosis was, however, listed first more often for patient vignettes of common diagnoses as compared to those of uncommon ones.

[1] Food and Drug Administration

[2] Health Insurance Portability and Accountability Act

[3] Not applicable

References:

24 - Bauer M, Glenn T, Monteith S, Bauer R, Whybrow PC, Geddes J. Ethical perspectives on recommending digital technology for patients with mental illness. Int J Bipolar Disord 2017 Dec; 5(1):6

25 - Weldegebrial T. Marshall University, Department of Health Informatics. 2016. Regulations for eHealth & mHealth Apps before Deployment [accessed 2019-04-06]

26- Bisson LJ, Komm JT, Bernas GA, Fineberg MS, Marzo JM, Rauh MA, Smolinski RJ, Wind WM. Accuracy of a computer-based diagnostic program for ambulatory patients with knee pain. Am J Sports Med 2014 Oct; 42(10):2371-6

27 - Bisson LJ, Komm JT, Bernas GA, Fineberg MS, Marzo JM, Rauh MA, Smolinski RJ, Wind WM. How Accurate Are Patients at Diagnosing the Cause of Their Knee Pain With the Help of a Web-based Symptom Checker?. Orthop J Sports Med 2016 Feb; 4(2):2325967116630286

28 - Boulos MNK, Brewer AC, Karimkhani C, Buller DB, Dellavalle RP. Mobile medical and health apps: state of the art, concerns, regulatory control and certification. Online J Public Health Inform 2014; 5(3):229

29 - Copeland C, Morreale P, Li J. M-Health Application Interface Design for Symptom Checking. 2018. Presented at: the 10th International Conference on e-Health; 2018; Madrid. p. 17-19

30 - Davies BM, Munro CF, Kotter MR. A Novel Insight Into the Challenges of Diagnosing Degenerative Cervical Myelopathy Using Web-Based Symptom Checkers. J Med Internet Res 2019 Jan 11; 21(1):e10868

31- Flaherty JL. Digital Diagnosis: Privacy and the Regulation of Mobile Phone Health Applications. Am J Law Med 2014; 40(4):416-41

32- Farmer SEJ, Bernardotto M, Singh V. How good is Internet self-diagnosis of ENT symptoms using Boots WebMD symptom checker?. Clin Otolaryngol 2011 Oct; 36(5):517-8

33 - Hageman MGJS, Anderson J, Blok R, Bossen JKJ, Ring D. Internet self-diagnosis in hand surgery. Hand (N Y) 2015 Sept; 10(3):565-9

34 - Jutel A, Lupton D. Digitizing diagnosis: a review of mobile applications in the diagnostic process. Diagnosis (Berl) 2015 Jun 01; 2(2):89-96

35 - Kao C, Liebovitz DM. Consumer Mobile Health Apps: Current State, Barriers, and Future Directions. PM R 2017 May; 9(5S):S106-S115

36- Lanseng E, Andreassen T. Electronic healthcare: a study of people's readiness and attitude toward performing self-diagnosis. IJSIM 2007; 18(4):394-417

37 - Luger TM, Houston TK, Suls J. Older adult experience of online diagnosis: results from a scenario-based think-aloud protocol. J Med Internet Res 2014 Jan 16; 16(1):e16

38 - Lupton D, Jutel A. 'It's like having a physician in your pocket!' A critical analysis of self-diagnosis smartphone apps. Soc Sci Med 2015 May; 133:128-35

39 - Morita T, Rahman A, Hasegawa T, Ozaki A, Tanimoto T. The Potential Possibility of Symptom Checker. Int J Health Policy Manag 2017 Dec 05; 6(10):615-616

40- Powley L, McIlroy G, Simons G, Raza K. Are online symptoms checkers useful for patients with inflammatory arthritis? BMC Musculoskelet Disord 2016 Dec 24; 17(1):362

41- Ryan A, Wilson S. Internet healthcare: do self-diagnosis sites do more harm than good?. Expert Opin Drug Saf 2008 May; 7(3):227-9

42- Tudor Car L, Papachristou N, Bull A, Majeed A, Gallagher J, El-Khatib M, Aylin P, Rudan I, Atun R, Car J, Vincent C. Clinician-identified problems and solutions for delayed diagnosis in primary care: a PRIORITIZE study. BMC Fam Pract 2016 Dec 09; 17:131
